# Spatial transcriptomics of the nematode *Caenorhabditis elegans* using RNA tomography

**DOI:** 10.1016/j.xpro.2021.100411

**Published:** 2021-03-30

**Authors:** Erik S. Schild, Jonas Mars, Annabel Ebbing, Judith Vivié, Marco Betist, Hendrik C. Korswagen

**Affiliations:** 1 Hubrecht Institute, Royal Netherlands Academy of Arts and Sciences and University Medical Center Utrecht, Uppsalalaan 8, 3584 CT Utrecht, the Netherlands; 2 Institute of Biodynamics and Biocomplexity, Developmental Biology, Department of Biology, Utrecht University, Padualaan 8, 3584 CH Utrecht, the Netherlands; 3 Single Cell Discoveries B.V., Uppsalalaan 8, 3584 CT Utrecht, the Netherlands

**Keywords:** Sequence analysis, RNA-seq, Model Organisms

## Abstract

RNA tomography or tomo-seq combines mRNA sequencing and cryo-sectioning to spatially resolve gene expression. We have adapted this method for the nematode *Caenorhabditis elegans* to generate anteroposterior gene expression maps at near-cellular resolution. Here, we provide a detailed overview of the method and present two approaches: one that includes RNA isolation for maximum sensitivity and one that is suitable for partial automatization and is therefore less time-consuming.

For complete details on the use and execution of this protocol, please refer to Ebbing et al. (2018).

## Highlights

• Spatial transcriptomics method combining cryo-sectioning with mRNA sequencing
• Separate protocols for maximal sensitivity versus partial automation
• Bioinformatic analysis pipeline for quality control of mRNA sequencing data
• Discussion of pitfalls and quality control issues

## Graphical Abstract

**Figure:**
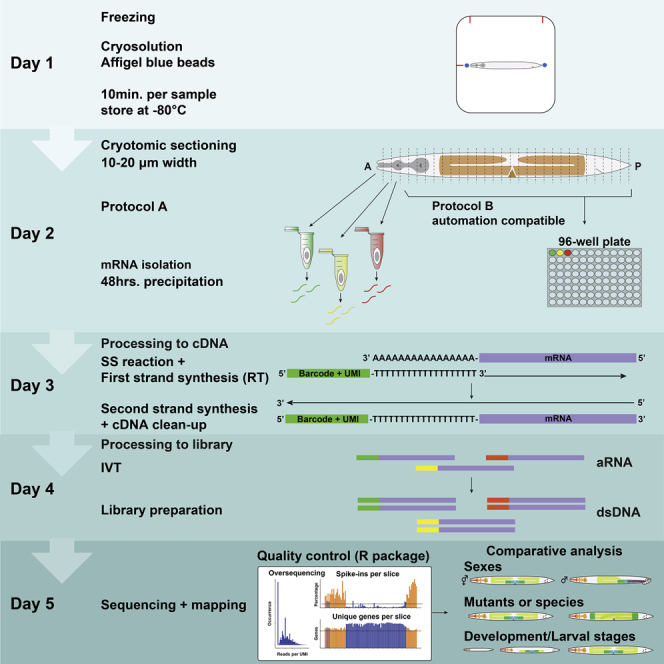

## Before you begin

***Note:*** We provide two different protocols, one with RNA isolation (protocol A) and one without (protocol B). In our experience, both approaches can produce high-quality libraries for sequencing, but there are some limitations that should be considered before starting the procedure. By first isolating the RNA, the input material will be more pure and smaller volumes of reagents can be used. However, the manual processing of large numbers of samples is labor intensive and may increase the chance of RNA degradation. Without RNA isolation, the hands-on time is much shorter, but the quality of the input material will be lower. For clarity, the parts of the method that are different between protocol A and B are described separately.

***Note:*** We recommend first testing the protocol with control RNA. There are several options available, such as HeLa-S3 RNA (Ambion).

### Staging and freezing of animals

**Timing for staging: depends on desired stage; hours to days**

**Timing for freezing: approximately 5 min per animal**

**Figure 1:**
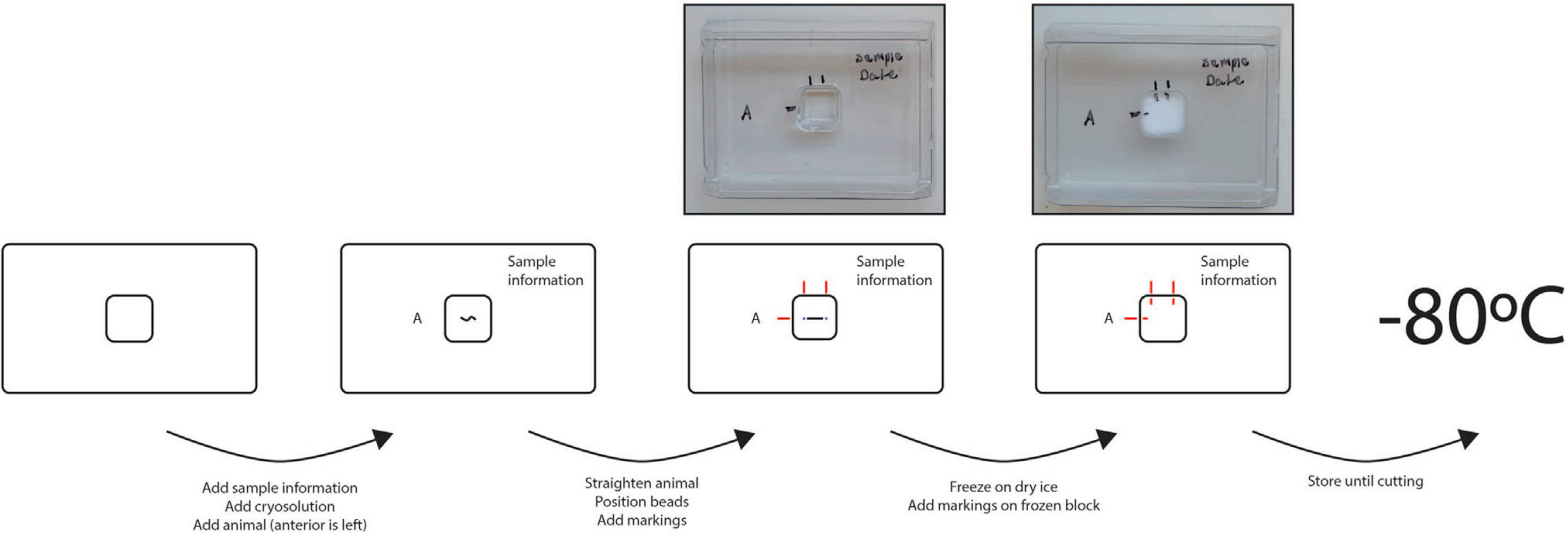
How to freeze and label an animal Add sample information to the base mold, then add cryo-solution and place the animal in the mold. Make sure to mark the anterior side of the animal on the base mold. Straighten the animal and position one Affi-Gel Blue Gel bead close to the anterior and the posterior of the animal. Add markings on the base mold that roughly indicate the locations of the beads. Quickly freeze the base mold on dry ice, ensuring the animal is straight. When frozen, extend the three markings to the frozen block. Store the cryo-block in the base mold at −80°C until cryosectioning.

1. Stage animals under desired conditions until they reach the appropriate stage.
2. Prepare freezing equipment:
  a. Dry ice
  b. Disposable Base Molds
  c. Tissue Freezing Medium
  d. Two eyelash picks
3. Pick animals into Tissue Freezing Medium in Disposable Base Mold (Figure 1).

**CRITICAL:** Animals should be frozen as quickly as possible. If more than 10 min are spent on one animal, discard this sample and repeat with another animal.

**CRITICAL:** Do not fill the Disposable Base Mold more than halfway with Tissue Freezing Medium, as this will interfere with later steps in the protocol.

**CRITICAL:** Ensure the animal is straight and not moving. A freeze-thaw cycle on dry ice may be needed to ensure the animal is motionless.

***Note:*** If very small animals are used (e.g., in the first larval stage), two animals may fit in one Disposable Base Mold, and 96 slices.

4. Position an Affi-Gel Blue Gel bead just anterior and posterior of the animal and mark the anterior of the animal and the positions of the beads on the Disposable Base Mold (Figure 1).
5. Quickly place the Disposable Base Mold on dry ice to freeze (Figure 1).
6. Extend the marks from the mold to the block (Figure 1). This will prevent confusion about the orientation of the animal when the block is taken out of the mold.

**Pause point:** Samples can be stored at −80°C. Storage can be extended up to a year; we have not tested longer storage.

## Key resources table

**Table:** 

REAGENT or RESOURCE	SOURCE	IDENTIFIER
**Bacterial and virus strains**		

OP50	Caenorhabditis Genetics Center	N/A

**Chemicals, peptides, and recombinant proteins**		

NGM Agar	Oxoid	CM1110
Tissue Freezing Medium	Leica Microsystems	14020108926
Affi-Gel® Blue Gel (100–200 mesh)	Bio-Rad Laboratories	CAT#1537302
Mineral oil	Sigma-Aldrich®	M8410-1L; CAS: 8042-47-5
TRIzol™ Reagent	Invitrogen™	CAT#15596018
Chloroform stabilized with ethanol	Boom	76050486.1000; CAS: 67-66-3
GlycoBlue™ Coprecipitant	Ambion®	CAT#AM9516
2-propanol, HPCL	Boom	75162606.2500; CAS: 67-63-0
Ethanol 100% absolute	Boom	840.281.852.500; CAS: 64-17-5
RNaseOUT	Invitrogen™	CAT#10777-019
H_2_O Nuclease free	Invitrogen™	CAT#10793837
Second strand buffer	Invitrogen™	CAT#10812-014
*E. coli* DNA ligase	Invitrogen™	CAT#18052-019
*E. coli* DNA polymerase I	Invitrogen™	CAT#18010-025
*E. coli* RnaseH	Invitrogen™	CAT#18021-071
PEG8000	Sigma-Aldrich®	1546605; CAS: 25322-68-3
ExoSAP-IT™ PCR Product Cleanup Reagent	Invitrogen™	78201.1.ML
KOAc	Sigma-Aldrich®	95843; CAS: 127-08-2
MgOAc	Sigma-Aldrich®	63052; CAS: 142-72-3
Ethylenediaminetetraacetic acid solution pH 8	Sigma-Aldrich®	03690; CAS: 60-00-4
TruSeq small RNA sample prep kit (containing both the RPIX and RP1 primers)	Illumina®	RS-200-0012

**Critical commercial assays**		

ERCC RNA Spike-In Mix	Ambion®	CAT#4456740
CEL-Seq2 primers	Integrated DNA Technologies IDT	N/A
SuperScript II Reverse Transcriptase kit	Invitrogen™	CAT#18064-014
Deoxynucleotide (dNTP) Mix	Promega	CAT#U1515
AMPure XP Beads	Beckman Coulter A63880	CAT#NC9959336
MEGAscript T7 Transcription Kit	Ambion®	CAT#AM1334
RNAClean XP Beads	Beckman Coulter	CAT#A63987
RNA 6000 pico kit	Agilent Technologies	PART#5067-1513
randomhexRT primers (sequence 5’-GCC TTG GCA CCC GAG AAT TCC A(N:25252525)(N)(N)(N)(N)(N)-3’)	Integrated DNA Technologies IDT	N/A
Qubit dsDNA HS Assay Kit	Invitrogen™	CAT#Q32854
High sensitivity DNA kit	Agilent Technologies	CAT#5067-4626
High-fidelity 2× PCR Master Mix	NEB Inc.	CAT#M0541 (S or L)

**Experimental models: organisms/strains**		

*C. elegans*: wild-type N2	Caenorhabditis Genetics Center (CGC)	WB Strain: N2

**Software and algorithms**		

BWA v0.7.10	Li and Durbin (2010). PMID: 20080505	http://bio-bwa.sourceforge.net/
MapAndGo v2	This paper	http://bit.ly/MapAndGo_v2
R (v 4.0.3), using the packages: TomoQC	This paper	https://cran.rproject.org/, https://github.com/erikschild/TomoQC

**Other**		

Fisher Healthcare™ Disposable Base Molds	Invitrogen™	CAT#22-363-553
LoBind Tubes 0.5 mL	Eppendorf Tubes®	ITEM#EP0030108035
Hard-shell PCR low-profile, semi-skirted 96-well plate	Bio-Rad Laboratories	HSL9601
Jewelers forceps, Dumont No. 5	Sigma-Aldrich®	F6521; eCl@ss: 32035504
Block heater, analogue	VWR™	460-0355
Enkele blok PCR-buizen (0,5 mL)	VWR™	460-3211
LoBind tubes (1.5 mL)	Eppendorf Tubes®	ITEM#EP0030108051
LoBind tubes (5.0 mL)	Eppendorf Tubes®	ITEM#EP0030122348
DynaMag 2	Invitrogen™	CAT#12321D
SILVERseal sealer, Aluminium	Greiner Bio-One	SKU#676090

## Materials and equipment

**Table:** Bead binding buffer

Reagent	Final concentration	Stock concentration	Add to 15 mL
PEG8000	20%	100%	3 mL
NaCl	2.5M	5M	7.5 mL
Nuclease-free water			4.5 mL

Store bead binding buffer at 20°C–25°C.

**Table:** Amplified RNA (aRNA) fragmentation buffer

Reagent	Final concentration	Stock concentration	Add to 5.5 μL
Tris-acetate [pH 8.1]	40 mM	200 mM	1.1 μL
KOAc	500 mM	5 M	0.55 μL
MgOAc	150 mM	1 M	0.85 μL
Nuclease-free water			3 μL

Store aRNA fragmentation buffer at 20°C–25°C.

**Table:** Fragmentation STOP buffer

Reagent	Final concentration	Stock concentration	Add to 2.75 μL
EDTA [pH 8.0]	20%	100%	0.55 μL
Nuclease-free water			2.2 μL

Store fragmentation STOP buffer at 20°C–25°C.

## Step-by-step method details

### Protocol A: Tomo-sequencing including RNA isolation

#### Prepare collection tubes

**Timing: 5 min**

1. Number 96 0.5 mL microcentrifuge tubes.

#### Sectioning and collection of frozen animals

**Timing: 45 min per animal**

The frozen sample is sectioned in a cryostat (ThermoFisher CryoStar NX70) and each slice is collected for further processing. The separate processing of each slice is the critical step in providing spatial information to the resulting sequencing library.

2. Prepare freezing equipment (Figure 2):
  a. Dry ice
  b. Two small brushes (keep at −20°C)
  c. Forceps (keep at −20°C)
  d. Numbered 0.5 mL microcentrifuge tubes (keep on dry ice)
  e. Tissue Freezing Medium (keep at RT)
  f. Sample(s) to slice (keep on dry ice)

**Figure 2:**
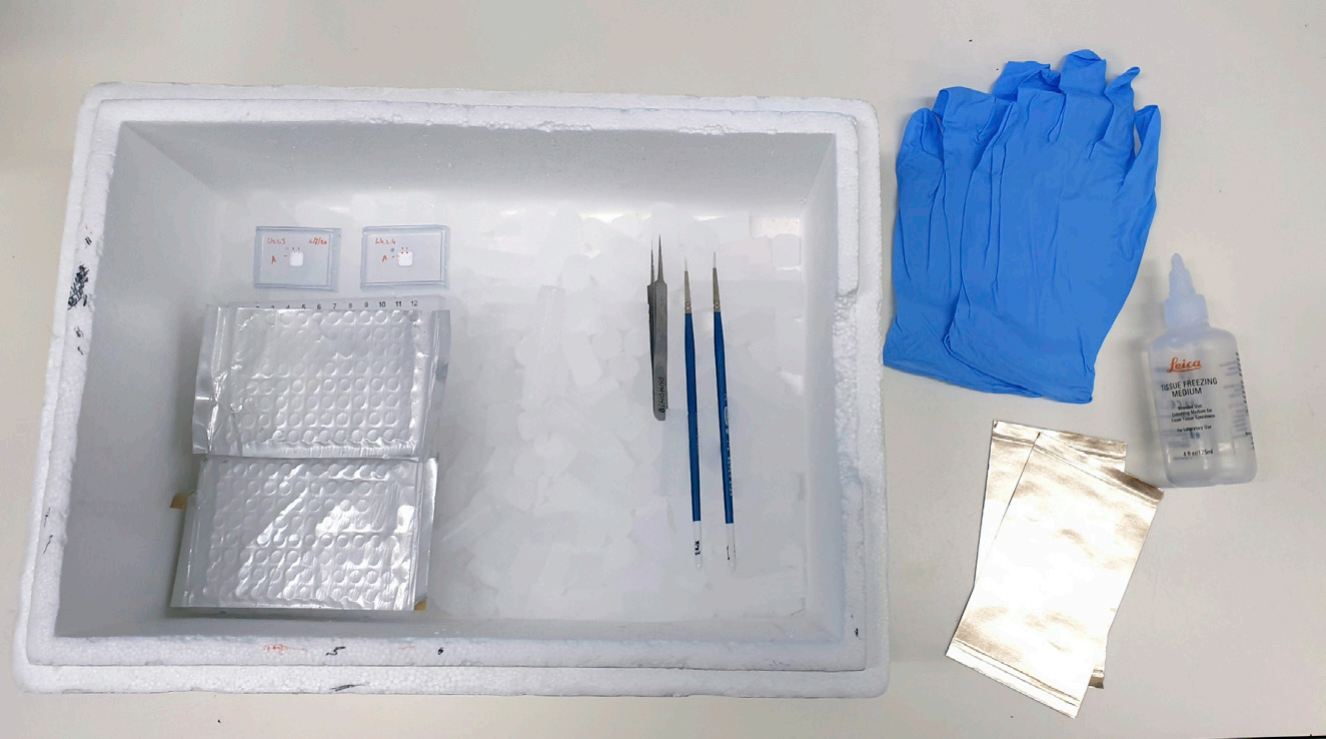
Materials required for cutting sample From left to right: a box with dry ice, containing the base mold containing the sample cryo-block; 96-well plates for slice collection; a forceps and two brushes. Gloves, sealing film and cryo-solution are also required. Note that the 96-well plates are required for protocol B; protocol A uses 96 labeled 0.5 mL tubes.
3. Apply a generous amount (approximately 2 mL) of Tissue Freezing Medium to a cryostat specimen disc and place it in the cryostat (Figure 3).

**Figure 3:**
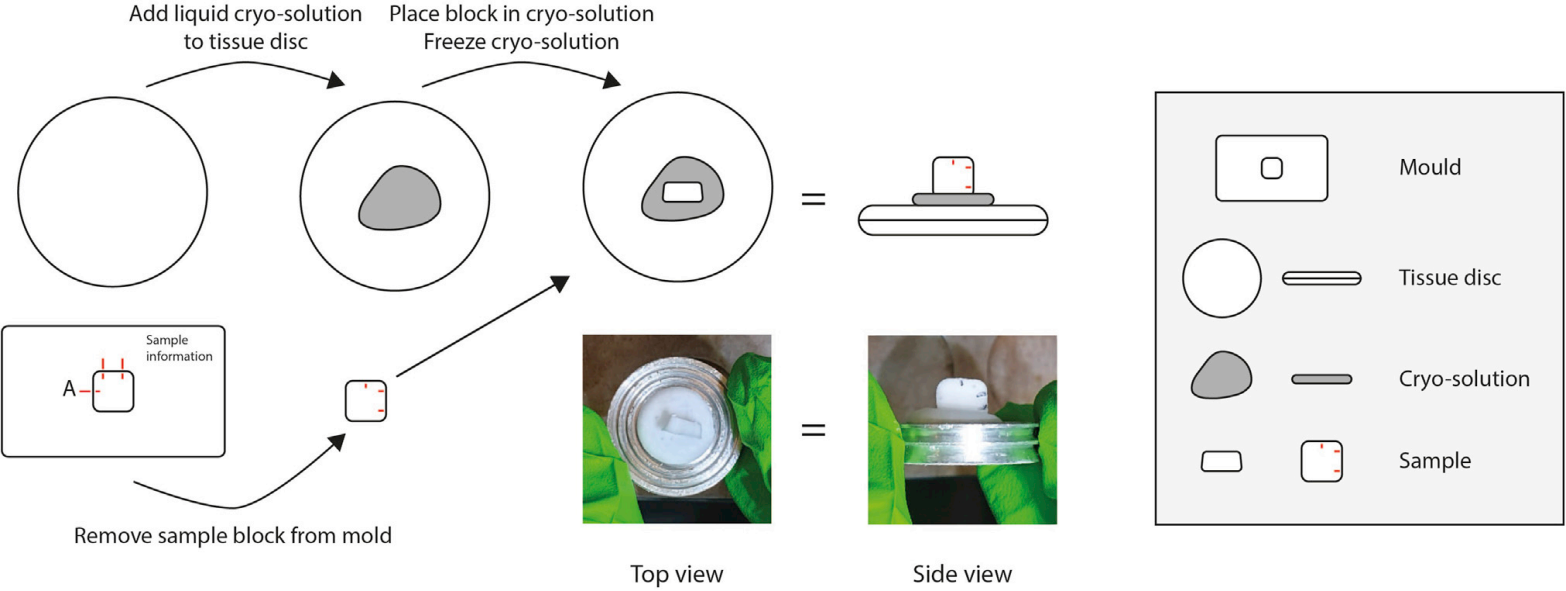
How to prepare sample for cryosectioning Remove the sample cryo-block from the base mold and place it on the tissue disc containing cryo-solution, with the anterior facing upwards. Wait until the cryo-solution is completely frozen before starting cryosectioning.
4. Using forceps, position the sample block in the (not yet frozen) Tissue Freezing Medium, so that the anterior of the animal faces up from the specimen disc (Figure 3).
5. Wait until the block is completely frozen and attached to the specimen disc, then place the specimen disc in the sample holder (Figure 3).

***Note:*** If necessary, adjust the angle of the sample holder so that it will cut perpendicular to the anteroposterior axis of the worm.

6. Cut thick slices of 50–100 μm until the first blue bead appears, then switch to desired slice thickness. A 20 μm slice of a young adult hermaphrodite contains an average of 25 cells.

***Note:*** In our experience, the minimal thickness for sectioning larvae and adult animals is 10 μm. We have sectioned animals at various thicknesses between 10 μm and 20 μm.

***Note:*** You can use the marks on the block as a rough indication of when to expect the first bead.

**CRITICAL:** Trim the cryo-block on the sides to limit the amount of frozen cryo-solution that is collected. Roughly 2 mm space on all four sides of the bead is sufficient to ensure the whole animal is present.

7. Start collecting slices using brushes and forceps. When the first bead becomes fainter, place them in the labeled 0.5 mL microcentrifuge tubes: slice 1 in tube 1, slice 2 in tube 2, et cetera, until the posterior bead appears (Methods video S1. Sectioning and collection of frozen animals).

***Note:*** Individual slices should be transferred to microcentrifuge tubes and placed on dry ice as quickly as possible, to minimize the chance of RNA degradation.

***Note:*** When the posterior bead appears, write down the total number of slices for later reference.

***Note:*** Based on the length of the animal and the sectioning width, the number of slices that span the distance between the blue beads can be estimated. If the actual number of slices is significantly lower, this indicates that the bead has moved during the freezing process. If so, discard the sample.

Methods video S1. Sectioning and collection of frozen animals, related to steps A7 and B8 The frozen sample is sectioned in a cryostat (ThermoFisher CryoStar NX70) and each slice is collected for further processing.

#### RNA extraction

**Timing: 2 h + 48 h of incubation time**

The frozen sections are further processed to isolate the RNA. We add Glycoblue to help visualize the RNA pellets, and ERCC spike-ins as a quality control measure downstream in the protocol.

8. Add 100 μL of the following mixture to each microcentrifuge tube containing a slice, and shake vigorously for 5 s:

**Table:** 

Trizol	100 μL
ERCC Spike-ins (diluted to 1:500.000 in nuclease-free water)	0.1 μL

9. Spin the samples down at 12.000 *g* and 4°C, for a few seconds.
10. Add 20 μL of chloroform to each microcentrifuge tube and shake vigorously for 5 s.
11. Spin the samples down at 12.000 *g* and 4°C, for 15 min.

Number as many 0.5 mL microcentrifuge tubes as you have slices. Add 0.2 μL of Glycoblue (15 mg/mL) to each tube.

***Note:*** The addition of Glycoblue helps visualization of the pellet.

12. Transfer about 60 μL of the upper aqueous layer to the new, numbered 0.5 mL microcentrifuge tubes containing 0.2 μL of Glycoblue, add 60 μL of isopropanol and shake vigorously for 5 s.

**Pause point:** Store the samples for at least 48 h at −20°C. The longer the storage time, the higher the final RNA yield.

13. Spin the samples down at 12.000 *g* and 4°C, for 18 min.
14. Aspirate supernatant carefully.

***Note:*** Use pipettes with smaller tips to ensure complete removal of the supernatant without disturbing the pellet.

15. Add 180 μL of freshly diluted 75% ethanol (20°C–25°C) to each sample, vortex and spin down at 7.500 *g* and 4°C, for 10 min.
16. Aspirate supernatant, add 180 μL of 75% ethanol (20°C–25°C) to each sample, vortex and spin down at 7.500 *g* and 4°C, for 10 min.
17. Aspirate the supernatant, and remove as many excess ethanol droplets as possible using a 2 μL pipette.
18. Air dry the pellet until completely dry, for a maximum of 10 min.

**CRITICAL:** Prolonged drying will result in degradation of the RNA and makes it more difficult to dissolve the pellet. One will need to find the perfect timing at which the pellet is dry, but the drying time is as short as possible. Therefore, removing as many ethanol droplets as possible in step 18 is crucial.

#### RNA amplification

**Timing: 15 min**

**Figure 4:**
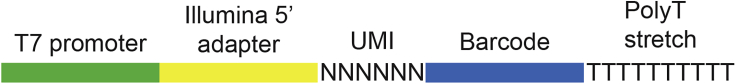
Components of the CelSeq2 primer The CelSeq2 primer contains a T7 promoter element, an Illumina 5’ adapter, a unique molecular identifier (6 bp), a cell-specific barcode (8 bp) and a poly-T stretch.

In single-cell mRNA sequencing, amplification of RNA is not trivial. Novel methods often rely on the use of an *in vitro* transcription step (CEL-Seq) (Hashimshony et al., 2012), in combination with an unique molecular identifier (UMI) to detect unique reads (Grün et al., 2014), referred to as CEL-Seq2 (Hashimshony et al., 2016). The principle relies on the use of individual RNA annealing primers that contains a T7 promoter, Illumina 5’ adapter, UMI, cell-specific barcode and poly-T-sequence (Figure 4). The UMI (4 bp) and barcode (6 bp) can be traced back from the sequencing data to distinguish reads from individual cells.

We make use of an adapted version of the CEL-Seq2 protocol, that will label each detected molecule with a section-specific barcode (8 bp) (Table S1) and UMI (6 bp). By adding unique CEL-Seq2 primers to the samples from consecutive cryo-sections, we are able to trace back reads from corresponding cryo-sections. This is the critical step in providing spatial information to the resulting sequencing library.

19. Resuspend the RNA pellet in 1 μL of the corresponding CEL-Seq2 primer (7.5 ng/μL) and keep the samples on ice until the next step.

**CRITICAL:** Ensure that the CEL-Seq2 primer number used for each sample corresponds to the slice number.

20. Incubate the samples for 1 min at 70°C in a heat block.

***Note:*** When only one heat block is available, change the temperature to 42°C directly after this step. This will speed up the next steps and therefore reduce the chance of RNA degradation.

21. Immediately move the samples back to ice and spin them down at 20.000 *g* for 10 s.

#### Reverse transcription

**Timing: 90 min**

Reverse transcription to cDNA:

22. Add 1 μL of the following mix to each microcentrifuge tube:

**Table:** 

First Strand Buffer (5×)	0.4 μL
DTT (0.1M)	0.2 μL
RNase Inhibitor (RNase OUT, 40 U/μL)	0.1 μL
SuperScriptII (200 U/μL)	0.1 μL
dNTPs (20 μM)	0.1 μL
Nuclease-free water	0.1 μL

23. Incubate the samples at 42°C, for 75 min in a heat block. Spin the samples down at 20.000 *g* for 10 s and move the samples back to ice.

#### Second strand reaction

**Timing: 90 min**

Generation of double-stranded cDNA:

24. Add 11 μL of the following mix to each microcentrifuge tube:

**Table:** 

Nuclease-Free Water	7.7 μL
Second strand buffer (5×)	2.5 μL
dNTPs (10 μM)	0.25 μL
*E. coli* DNA ligase (10 U/μL)	0.09 μL
*E. coli* DNA polymerase I (10 U/μL)	0.35 μL
*E. coli* RNAse H (2U/μL)	0.09 μL

***Note:*** As the samples are already barcoded, this step can be performed with the same tip as long as all samples will go into the same IVT reaction.

25. Gently flick samples and spin them down for a few seconds, then incubate the samples at 16°C for 75 min.

#### cDNA cleanup using magnetic beads

**Timing: 1 h**

The cDNA is cleaned and eluted in nuclease-free water.

26. Warm AMPure XP beads to room temperature (20°C–25°C).
27. Pool all reactions that will go into the same IVT reaction into one 1.5 mL microcentrifuge tube.
28. Prepare bead mix in a 1:7 ratio of beads to bead binding buffer, vortex well and add the bead mix to the sample in a 4:5 ratio (80 μL bead mix for 100 μL cDNA).
29. Incubate the sample at room temperature (20°C–25°C) for 15 min.
30. Place the sample on a magnetic stand for at least 5 min, until the liquid is clear.
31. Remove and discard the supernatant, add 800 μL of freshly diluted 80% ethanol and incubate minimally 30 s.

***Note:*** Make sure that the beads are not disturbed while removing supernatant. During this first step, it is best to remove only part of the supernatant.

32. Repeat the wash step; then, remove all supernatant without disturbing the beads.
33. Air dry the pellet until completely dry, for a maximum of 10 min.
34. Resuspend the beads in 8 μL nuclease-free water. Pipette up and down 10 times to mix thoroughly.
35. Incubate at room temperature (20°C–25°C) for 2 min, then place the sample on the magnetic stand until the liquid is clear.
36. Carefully pipette all supernatant into a new 0.5 mL microcentrifuge tube, without disturbing the beads.

***Note:*** Spinning down the microcentrifuge tube and replacing it on the magnetic stand may be necessary to obtain all supernatant.

***Note:*** The original CEL-Seq2 protocol (Hashimshony et al., 2016) removes the AMPure XP beads after amplified RNA (aRNA) fragmentation (see below). Our alternative method, described here, is to remove the AMPure XP beads directly after cDNA cleanup. We have obtained good results with this approach (Table S2) and have not extensively tested the original CEL-Seq2 method.

**Pause point:** The sample can be stored at −20°C for one week.

***Note:*** After cDNA cleanup, continue with the IVT reaction as described in “in vitro transcription.”

### Protocol B: Tomo-sequencing without RNA isolation

#### Prepare 96-well plate for collecting slices

**Timing: 15 min**

In single-cell mRNA sequencing, amplification of RNA is not trivial. Novel methods often rely on the use of an *in vitro* transcription step (CEL-Seq) (Hashimshony et al., 2012), in combination with an unique molecular identifier (UMI) to detect unique reads (Grün, Kester and Van Oudenaarden, 2014), referred to as CEL-Seq2 (Hashimshony et al., 2016). The principle relies on the use of individual RNA annealing primers that contains a T7 promoter, Illumina 5’ adapter, UMI, cell-specific barcode and poly-T-sequence (Figure 4). The UMI (4 bp) and barcode (6 bp) can be traced back from the sequencing data to distinguish reads from individual cells.

We make use of an adapted version of the CEL-Seq2 protocol, that will label each detected molecule with a section-specific barcode (8 bp) (Table S1) and UMI (6 bp). By adding consecutive CEL-Seq2 primers to the samples from consecutive cryo-sections, we are able to trace back reads from corresponding cryo-sections. This is the critical step in providing spatial information to the resulting sequencing library.

1. Add 25 μL of mineral oil to each well. The oil prevents evaporation of the small reaction volumes.
2. Add 1 μL of individual CEL-Seq2 primer to each well and carefully film the plate.

**CRITICAL:** Ensure that the CEL-Seq2 primer number used for each sample corresponds to the slice number.

**Pause point:** Store the 96-well plate at −80°C. Storage can be extended to at least 3 months; we have not tested longer storage.

#### Sectioning and collection of frozen animals

**Timing: 45 min per animal**

Animals frozen in blocks earlier are cryo-sectioned in a cryostat (ThermoFisher CryoStar NX70) and slices are collected for further processing. The separate processing of each slice is the critical step in providing spatial information to the resulting sequencing library.

3. Prepare freezing equipment (Figure 2):
  a. Dry ice
  b. Two small brushes (keep at −20°C)
  c. Forceps (keep at −20°C)
  d. The prepared 96-well plate (keep on dry ice)
  e. Tissue Freezing Medium (keep at RT)
  f. Sample(s) to slice (keep on dry ice)
4. Apply a generous amount (approximately 2 mL) of Tissue Freezing Medium to a cryostat specimen disc and place it in the cryostat.
5. Using forceps, position the sample block in the (not yet frozen) Tissue Freezing Medium, so that the anterior of the animal faces up from the specimen disc.
6. Wait until the block is completely frozen and attached to the specimen disc, then place the specimen disc in the sample holder.

***Note:*** If necessary, adjust the angle of the sample holder so that it will cut perpendicular to the anteroposterior axis of the animal.

7. Cut thick slices of about 50–100 μm until the first bead appears, then switch to desired slice thickness.

***Note:*** In our experience, the minimal thickness for sectioning larvae and adult animals is 10 μm. We have sectioned animals at various thicknesses between 10 μm and 20 μm.

***Note:*** You can use the marks on the block as a rough indication of when to expect the first bead.

**CRITICAL:** Trim the cryo-block on the sides to limit the amount of frozen cryo-solution that is collected. Roughly 2 mm space on all four sides of the bead is sufficient to ensure the whole animal is present. Failure to remove excess cryo-solution will seriously compromise library generation.

8. Start collecting slices using brushes and forceps. When the first bead becomes fainter, place the sections in the 96-well plate: slice 1 in well A1, slice 2 in well A2, et cetera, until the posterior bead appears (Methods video S1).
9. Carefully seal the plate with a 96-well plate sealing film.

**CRITICAL:** Sections may stay at the top of their well due to static electricity. For this reason, ensure sealing the plate occurs in one motion. A section may melt onto the sticker when applying, thus repositioning the film after application could cause a section to be lost or transferred to a different position.

**CRITICAL:** Do not touch the mineral oil with the forceps, as this might cause contamination between the wells.

***Note:*** Collecting in small batches is recommended. Make sure to place the 96-well plate back on dry ice as quickly as possible, to minimize degradation of RNA in the slices.

***Note:*** When the posterior bead appears, note the total number of slices used for reference later.

***Note:*** Based on the length of the animal and the sectioning width, the number of slices that span the distance between the blue beads can be estimated. If the actual number of slices is significantly lower, this indicates that the bead has moved during the freezing process. If so, discard the sample.

**Pause point:** Store the samples at −80°C. We generally continue the protocol within 24 h and have not tested longer storage.

#### RNA amplification

**Timing: 15 min**

10. Incubate the plate for 5 min at 65°C in a thermal cycler.
11. Move the plate back to ice and spin it down at 20.000 *g* for 10 s.

#### Reverse transcription

**Timing: 90 min**

The RNA is reverse transcribed to cDNA.

12. Add 1 μL of the following mix to each well:

**Table:** 

First Strand Buffer (5×)	0.4 μL
DTT (0.1M)	0.2 μL
RNase Inhibitor (RNase OUT, 40 U/μL)	0.1 μL
SuperScriptII (200 U/μL)	0.1 μL
dNTPs (20 μM)	0.1 μL
ERCC spike-ins (diluted to 1:50.000.000 in nuclease-free water)	0.1 μL

***Note:*** The reverse transcription reagents can also be dispensed by a robot. We have used the Nanodrop II (GC biotech). If this is the method of choice, add dNTPs (20 μM) and ERCC spike-ins (diluted to 1:50.000.000) to the 96-well plate before the heat lysis step as described in step 9. Continue with step 10 above and add the remaining reagents for step 12. Continue the reverse transcription reaction as described below in step 13.

13. Incubate the plate at 42°C, for 75 min in a thermal cycler and move the samples back to ice.

#### Second strand reaction

**Timing: 90 min**

To produce double-stranded cDNA.

14. Add 11 μL of the following mix to each well:

**Table:** 

Nuclease-free water	7.7 μL
Second strand buffer (5×)	2.5 μL
dNTPs (10 μM)	0.25 μL
*E. coli* DNA ligase (10 U/μL)	0.09 μL
*E. coli* DNA polymerase I (10 U/μL)	0.35 μL
*E. coli* RNAse H (2U/μL)	0.09 μL

***Note:*** As the samples are already barcoded, this step can be performed with the same tip. Only if more than one animal is processed at the same time, do not use the same tip for separate samples.

***Note:*** We mix the second strand reaction reagents by hand. Alternatively, this could be dispensed by the Nanodrop II (GC biotech) or other 8-channel pipetting robots, but we have not tested this.

15. Gently flick and tap the plate, spin it down for a few seconds, then incubate at 16°C for 75 min.

#### cDNA purification using magnetic beads

**Timing: 1 h**

The cDNA is cleaned and eluted in nuclease-free water.

16. Warm AMPure XP beads to room temperature (20°C–25°C).
17. Pool all reactions into one tube, spin down for 1 min at 12.000 *g* and transfer the lower, aqueous phase to a new microcentrifuge tube.

***Note:*** If more than one animal is processed at the same time, pool all reactions that belong to the same animal.

***Note:*** Pooling can be done in several ways. We have experienced good results by centrifuging the plate upside down at a low speed (1 min at 250 *g*), thereby collecting all samples into one container. Subsequently, all volume can be transferred to a 5 mL tube and spun down (1 min at 12.000 *g*) to separate the aqueous phase to transfer to a new 1.5 mL tube. Alternatively, one could pool all reactions into 1.5 mL tubes directly and spin down to separate the aqueous phase. However, due to the total volume exceeding 1.5 mL, multiple tubes will be necessary if more than 35 sections are used.

***Note:*** There will always be some residual mineral oil left in the transferred aqueous phase. Therefore, the volume will be higher than the number of samples multiplied by 13 μL of cDNA. Moreover, if a full 96-well plate has been used (96 slices), the bead cleanup cannot be performed in one 1.5 mL tube. If this is this case, we recommend splitting the sample, and performing 2 parallel bead cleanups in separate 1.5 mL tubes, up until elution. Since the final elution volume must be 8 μL, we recommend using 8 μL of nuclease-free water to elute the DNA from the beads in one tube, then transfer this water to the other tube to elute the remaining DNA there.

18. Prepare bead mix in a 1:7 ratio of beads to bead binding buffer, vortex well and add the bead mix to the sample in a 4:5 ratio (80 μL bead mix for 100 μL cDNA).
19. Incubate the sample at room temperature (20°C–25°C) for 15 min.
20. Place the sample on a magnetic stand for at least 5 min, until the liquid is clear.
21. Remove and discard the supernatant, add 800 μL of fresh 80% ethanol and incubate minimally for 30 s.

***Note:*** Make sure that the beads are not disturbed while removing supernatant. During this first step, it is best to remove only part of the supernatant.

22. Repeat the wash step; then, remove all supernatant without disturbing the beads.
23. Air dry the pellet for a maximum of 10 min.

***Note:*** Residual mineral oil will always be in the sample and therefore, the pellet will never appear dry because of the oil layer. We advise to dry the beads for approximately 6–7 min to avoid over-drying the beads, which will make elution more difficult.

24. Resuspend the beads in 8 μL nuclease-free water. Pipette up-and-down 10 times to mix thoroughly.
25. Incubate at room temperature (20°C–25°C) for 2 min, then place the sample on the magnetic stand until the liquid is clear.
26. Carefully pipette all supernatant into a new 0.5 mL microcentrifuge tube, without disturbing the beads.

***Note:*** Spinning down the microcentrifuge tube and replacing it on the magnetic stand may be necessary to obtain all supernatant.

**Pause point:** The sample can be stored at −20°C for one week.

***Note:*** After cDNA cleanup, continue with the IVT reaction as described in “in vitro transcription.”

### Continuation of both Protocol A & B

The following sections describe the steps that should be taken to generate a cDNA library for sequencing and mapping. First, we amplify the purified cDNA using linear amplification to aRNA. Next, we reverse transcribe the aRNA to a cDNA library.

***Note:*** From here, all steps are identical for both tomo-sequencing with and without RNA isolation. The starting point is an 8 μL cDNA sample that has been stored at −20°C for less than one week.

***Optional:*** The next steps – until sequencing – can be automated using a pipetting robot.

#### *In vitro* transcription

**Timing: 15–20 h**

To transcribe the amplified cDNA to RNA.

***Note:****In vitro* transcription and library preparation are performed according to the CEL-Seq2 protocol (Hashimshony et al., 2016).

1. Prepare the following master mix (using the Ambion MegaScript T7 transcription kit) and add 12 μL to the 8 μL of eluted cDNA:

**Table:** 

ATPs	2 μL
GTPs	2 μL
CTPs	2 μL
UTPs	2 μL
T7 buffer 10×	2 μL
T7 enzyme	2 μL

2. Incubate the sample in a thermal cycler at 37°C for 13 h, with the lid set at 70°C. Set the cycler to go to 4°C at the end of the incubation period.

***Note:*** aRNA is stable for at least 6 h at 4°C, but we have not tested longer storage. aRNA can be stored for one week at −20°C.

3. Add 7.5 μL of EXO-SAP enzyme and incubate at 37°C for 15 min, to remove the CEL-Seq2 primers.
4. Mix all of the aRNA (27.5 μL) with 6.8 μL fragmentation buffer on ice, incubate at 94°C for 3 min and immediately move the sample to ice.

**CRITICAL:** Fragmenting the aRNA for longer than 3 min can significantly decrease the sample quality.

5. Add 3.5 μL fragmentation STOP buffer.
6. Prewarm RNAClean XP beads to room temperature (20°C–25°C) and vortex until well dispersed.
7. Add 60 μL beads to the sample and incubate at room temperature (20°C–25°C) for 10 min.
8. Place the sample on a magnetic stand for at least 5 min, until the liquid appears clear.
9. Remove and discard about 80 μL of the supernatant.
10. Add 200 μL fresh 70% ethanol, incubate for at least 30 s, then remove and discard the supernatant without disturbing the beads.
11. Repeat step 10 (washing) two more times.
12. Air dry the beads for a maximum of 10 min, or until they are completely dry.
13. Resuspend the beads with 7 μL nuclease-free water. Pipette the entire volume up and down ten times to mix thoroughly.
14. Incubate at room temperature (20°C–25°C) for 2 min, place on the magnetic stand for 5 min until the liquid appears clear. Then, transfer the supernatant to a new 0.5 mL microcentrifuge tube.

**Pause point:** Samples can be kept at −20°C for at least 2 months; we have not tested longer storage.

**Figure 5:**
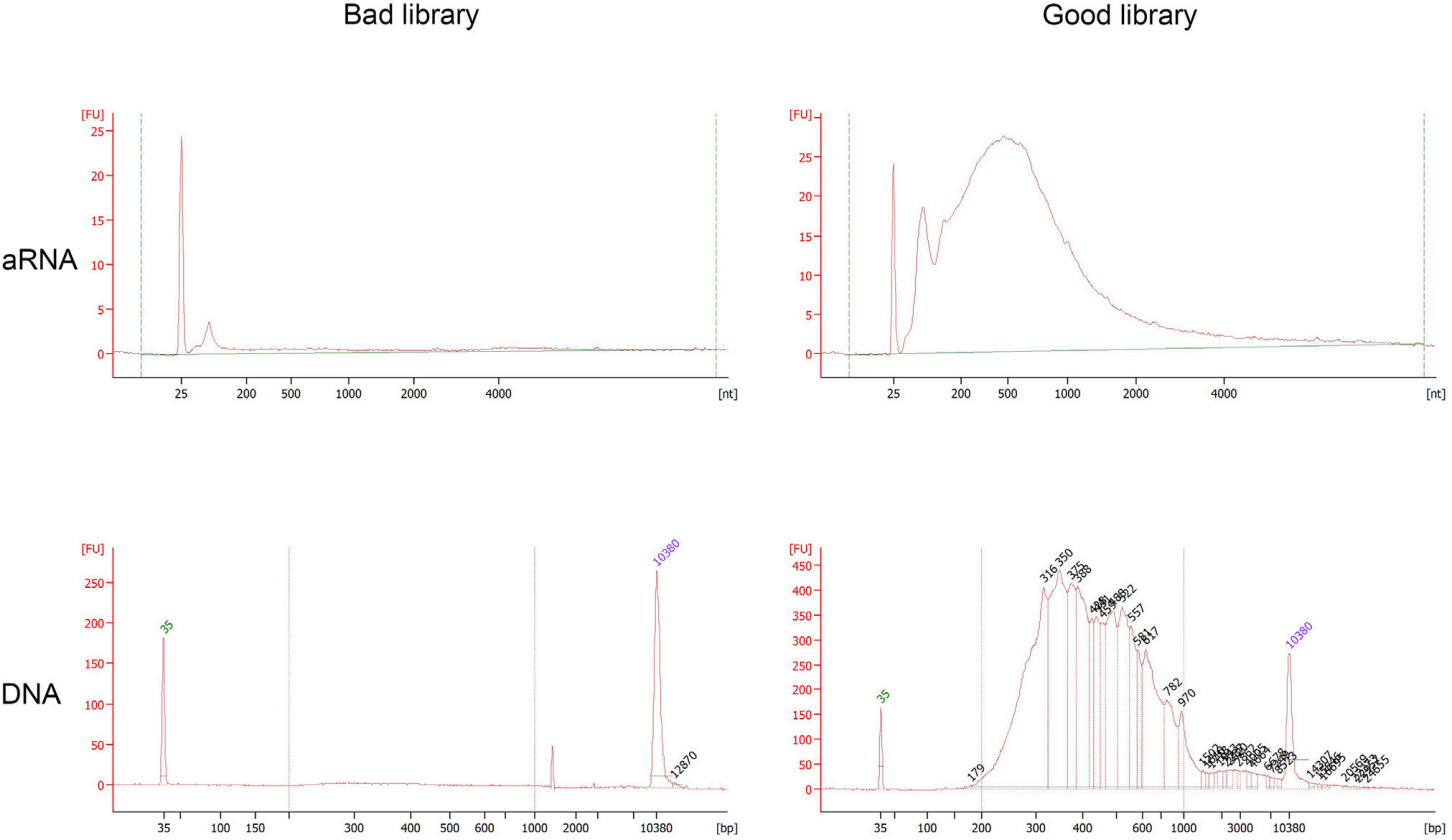
Example of bioAnalyzer outcomes Top: bioAnalyzer aRNA output, for a bad library (left) and a good library (right). Fragment sizes are expected to range from 50–1000 nucleotides. Bottom: bioAnalyzer cDNA output, for a bad library (left) and a good library (right). The expected peak is a normal distribution between 200–1000 base pairs.

***Optional:*** Check the aRNA amount and quality by loading 1 μL of the sample onto a Bioanalyzer RNA pico chip. When the IVT reaction is started with approximately 0.1 ng RNA, the expected yield is 500 to 1000 pg/μL, with the bulk of fragment sizes ranging from 50–1000 nucleotides (Figure 5).

***Note:*** For very small samples, like 10 μm *C. elegans* sections, the aRNA content is not always visible on the Bioanalyzer. However, this is not necessarily an indication of failed amplification. Instead, perform the steps for library preparation after resuspending the beads in 5.5 μL instead of 7 μL nuclease-free water (as described in step 13).

#### Library preparation

**Timing: 3–5 h**

To prepare a DNA library of the amplified RNA sample.

To 5 μL of aRNA, add 1 μL random hexamer RT primers (20 μM) and 0.5 μL dNTPs (10 μM). We have the random hexRT primers custom-made at Integrated DNA Technologies (IDT) and order 100 μM of 100 nanomole hand-mixed DNA oligo at standard desalting (see key resources table). Incubate at 65°C for 5 min and place the sample on ice.

16. Prepare the following master mix at room temperature (20°C–25°C):

**Table:** 

First Strand Buffer (2×)	2.0 μL
DTT 0.1M	1.0 μL
RNase Inhibitor (RNase OUT, 40 U/μL)	0.5 μL
SuperScriptII (200U/l)	0.5 μL

17. Add 4 μL of this master mix to the sample and incubate for 10 min at 25°C, and subsequently for 1 h at 42°C.

***Note:*** Incubation at 42°C can be performed in a hybridization oven or, alternatively, in a pre-heated thermal cycler with the lid set at 50°C.

18. Add 40 μL of the following master mix to each sample:

**Table:** 

Nuclease-free water	11 μL
High-fidelity PCR master mix (2×)	25 μL
RNA PCR primer (RP1)	2 μL
A unique index RNA PCR primer (RPIX)	2 μL

**CRITICAL:** ensure that each sample has a uniquely indexed RNA PCR primer, as this is necessary to demultiplex separate samples after sequencing.

***Note:*** Both the RP1 and the RPIX primers are provided with the Illumina kit (Tables S3 and S4).

19. Amplify the cDNA sample in a thermal cycler with the following PCR conditions:
  a. 30 s at 98°C.
  b. 11 cycles of:
    i. 10 s at 98°C.
    ii. 30 s at 60°C.
    iii. 30 s at 72°C.
  c. 10 min at 72°C.
  d. Hold at 4°C.

***Note:*** Half of the PCR reaction is usually enough. However, if needed, the remaining 25 μL can be used to repeat the PCR amplification with more cycles.

***Note:*** If the aRNA concentration was low (see Figure 5), up to 15 cycles can be used.

**Pause point:** sample can be kept at −20°C.

20. Prewarm the AMPure XP beads to room temperature (20°C–25°C), vortex until well dispersed, then add 1:1 beads to the PCR reaction (either 50 μL or 25 μL if half the reaction was performed). Mix the entire volume thoroughly by pipetting up and down ten times.
21. Incubate the sample at room temperature (20°C–25°C) for 15 min, then place the sample on a magnetic stand for at least 5 min, until the liquid appears clear.
22. Remove and discard 95 μL (or 45 μL, in case of the 25 μL PCR reaction) of the supernatant.
23. Add 200 μL fresh 80% ethanol, incubate at least 30 s, then remove and discard the supernatant without disturbing the beads.
24. Repeat step 9 (wash) one more time, then air dry the beads for 15 min, or until completely dry.
25. Resuspend the beads in 25 μL nuclease-free water. Mix the entire volume thoroughly by pipetting up and down ten times.
26. Incubate the sample at room temperature (20°C–25°C) for 2 min, then place the sample on a magnetic stand for 5 min, until the liquid appears clear.
27. Transfer 25 μL of the supernatant to a new microcentrifuge tube.
28. Repeat steps 6–13, with the following adjustments: add 25 μL beads and elute in 10 μL nuclease-free water in the end.
29. Transfer the 10 μL sample to a new microcentrifuge tube.

***Note:*** The microcentrifuge tube now contains the DNA library.

30. Check the concentration of the DNA library (for example using Qubit).

***Note:*** 1 μL should be enough to measure the concentration. The expected concentration is at least 1 ng/μL.

31. Run 1 μL of the sample on a Bioanalyzer using a high-sensitivity DNA chip to see the size distribution of the DNA.

***Note:*** The expected peak is a normal distribution between 200–1000 base pairs (Figure 5).

#### Sequencing

**Timing: variable.**

***Note:*** We have consistently obtained good results using the Illumina NextSeq500 platform. We only have experience with this machine, but equivalent machines may also yield good results.

32. Dilute or mix samples for one run according to the requirements of your sequencing facility of choice. If you are sequencing a library for the first time, we recommend to use a minimum of 15 million reads in a run.
33. Sequence the mix of samples on an Illumina NextSeq500 high output platform with the following settings: 1 × 75 bp, divided 1:2 over read 1 and 2. This is sufficient to obtain good sequencing results.
  a. Read 1: 25nt. This read is mostly important to determine the UMI and CEL-Seq2 barcode sequences and therefore, a shorter read length is required.
  b. Read 2: 50nt. This read contains gene-specific information and therefore, a higher read length is required.

***Note:*** In case of the Illumina NextSeq500 platform, sequencing should produce 8 .fastq files containing Read 1 and 2 for each of the 4 sequencing lanes.

#### Mapping

**Timing: variable.**

Paired-end reads obtained by sequencing may be mapped to a reference transcriptome of *C. elegans*. In our studies, a reference transcriptome compiled from the WormBase *C. elegans* reference genome version WS249, supplemented with ERCC spike-ins, was used. This is available in standard format on GitHub: http://bit.ly/Reference_C_elegans.

Read alignment can be performed using the BWA-MEM algorithm (Li and Durbin, 2010). We use a custom wrapper of the algorithm, MapAndGo2 (available on GitHub, http://bit.ly/MapAndGo_v2).

## Expected outcomes

A successful tomo-sequencing experiment results in a genome-wide, spatial expression map of your sample (Table S2). The transcript count table contains the best approximation of expression, based on UMIs detected per gene (Grün et al., 2014). This map may be used for various analyses, depending on the aim of the project. Please refer to (Ebbing et al., 2018; Rödelsperger et al., 2021) for examples of downstream analysis.

## Limitations

### Strengths

The main strength of tomo-sequencing, which sets it apart from other approaches such as single cell mRNA sequencing, is that it provides detailed information on where genes are expressed in the organism. The spatial resolution of these gene expression maps is high: using a sectioning width of 10 μm, the resolution along the anteroposterior axis of *C. elegans* is sufficient to distinguish expression profiles of neighboring cells (Ebbing et al., 2018). Another aspect that makes this approach particularly powerful in *C. elegans* and other nematode species is the invariant morphology of the animals. This provides the unique opportunity to precisely align and compare expression maps to identify differences in gene expression patterns, for example between mutants or even between different nematode species (Rödelsperger et al., 2021).

### Limitations

The main limitation of tomo-sequencing of small samples such as *C. elegans* is sensitivity and noise. Since the amount of input material is limited (depending on the sectioning width, slices contain as few as 10 somatic cells), an amplification-based method is required for sequencing. We have previously shown that we can detect over 90% of genes that are found with bulk RNA sequencing (Ebbing et al., 2018). However, at the level of the individual sections, it can still be challenging to robustly detect lowly expressed genes. In such cases, it is helpful to have multiple expression maps, which can be compared and also be combined to increase sensitivity (Ebbing et al., 2018; Rödelsperger et al., 2021).

## Troubleshooting

Tomo-sequencing involves many steps, but sample quality can only be assessed at two stages: after preparation of the library (using the bioAnalyzer output, Figure 5), and after sequencing. Therefore, there are only limited options for troubleshooting. Two separate causes of a failed experiment can be distinguished: issues during cryo-sectioning, and loss and/or degradation of RNA. Loss or degradation of total RNA can be determined from a bioAnalyzer plot, which shows aRNA and cDNA quantity. A low quantity is often the result of RNA degradation during RNA isolation or sample cleanup. On the other hand, individually missing slices will only reveal themselves after sequencing and do not greatly impact the total RNA content of the sample, thus leaving no trace in bioAnalyzer plots.

### Problem 1

Gaps in expression maps

**Figure 6:**
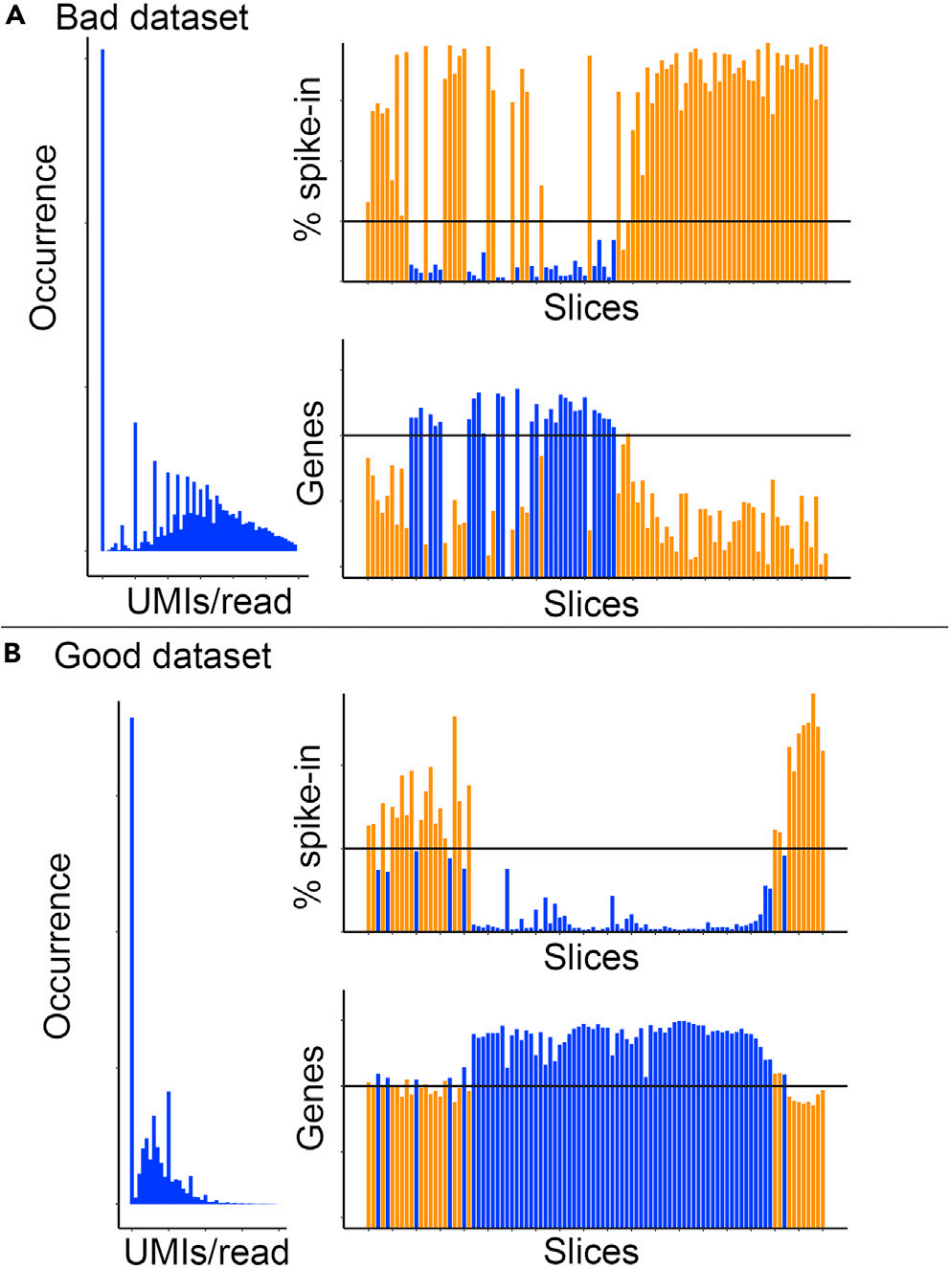
Example output of quality control R package Top: output for a bad dataset, with several missing slices and low complexity. Bottom: output for a good dataset, with high complexity.

After sequencing, the assembled expression map contains gaps (positions of sections that fall below the threshold of detected transcripts and genes) (Figure 6A).

### Potential solution 1

The most common causes for this are issues during cryo-sectioning or the individual RNA isolation steps, creating gaps in the expression map. In our experience, 10 μm is the minimal thickness to section *C. elegans* reliably. We offer a simple R package that distinguishes between columns of correctly sequenced sections from those that are not using two metrics: ‘unique gene count’ and ‘percentage spike-in reads’. The package is available at https://github.com/erikschild/TomoQC. When a slice is correctly sequenced, the unique gene count is expected to be higher than when there was no slice or the RNA was degraded. Conversely, the percentage of reads in the slice mapping to spike-ins will be much lower if the slice is correctly sequenced. Using customizable cutoffs, sections that contain sample can be distinguished from those that do not (Figure 6). Note that the package was designed as a quick overview of quality, not a precise tool to determine which columns correspond to the sample. Closer, manual inspection of the data is always necessary to select columns for further analysis.

### Problem 2

Low sample complexity

This problem is evident when shallow sequencing (e.g., 15M reads) results in many identical UMIs being detected per gene in a slice. This indicates that the sequencing finds many reads that do not represent separate endogenous mRNA molecules, but rather amplicons of a read, originating from one endogenous mRNA molecule. Seeing many of these reads means the library is oversequenced. Our aforementioned quality control R package plots the oversequencing state of the library (Figure 6). The further the peak in this plot shifts to the right, the closer to saturation/oversequencing the library is. Oversequencing is expected when sequencing deeply (45M reads is generally enough for saturation, but this depends on the size/age of the worm). When encountering oversequencing at (much) lower sequencing depth, it is likely the library has low complexity.

### Potential solution 2

Low complexity of a library cannot be solved after the fact. It is normally not possible to determine in what step RNA was degraded/lost leading to the low complexity. Repeating the experiment with a new sample is often the only recourse.

### Problem 3

Formation of a gel-like substance during cDNA bead cleanup (steps A34 and B24)

### Potential solution 3

If the bead mix, when combined with the cDNA sample, forms a gel-like substance, this is possibly due to the presence of polymers in the cryo-solution. This will significantly affect your cDNA library and further sample processing is impossible, as the gel-like substance prevents elution of the cDNA.

The issue will arise more often when relatively thick sections have been made (more than 20 μm). We encourage the researcher to always perform the following steps to avoid this problem: when the first blue bead becomes visible during sectioning, trim the cryo-block until a square-shaped block of approximately 2 × 2 mm is left, with the blue bead in the middle. Continue the protocol as normal.

## Resource availability

### Lead contact

Further information and requests for resources and reagents should be directed to and will be fulfilled by the lead contact, Prof. Dr. Hendrik C. Korswagen (r.korswagen@hubrecht.eu).

### Materials availability

This study did not generate new unique reagents.

### Data and code availability

The reference transcriptome of *C. elegans* used in this study is available on GitHub:

https://github.com/vertesy/TheCorvinas/tree/master/Biology/Sequencing/C_elegans

The custom wrapper for BWA mapping used in this study is available on GitHub:

https://github.com/vertesy/TheCorvinas/tree/master/Python/MapAndGo2

The quality control R package made for this study is available on GitHub:

https://github.com/erikschild/TomoQC

Data from an earlier study comparing *C. elegans* hermaphrodites and males using this protocol (Ebbing et al., 2018), is available here: http://celegans.tomoseq.genomes.nl/

## Supplemental information

Table S1. CelSeq2 primers used in this study, related to steps A19 and B2 The 384 CelSeq2 primers and their corresponding 8 bp barcodes.

Table S2. Example transcripts output mapping, related to expected outcomes An example of the transcripts output of the MapAndGo wrapper for BWA MEM mapping of a young adult hermaphrodite sample. Note that the output generated by MapAndGo is normally a tab delimited (.tsv) file, not an excel file (.xlsx).

Table S3. Illumina TruSeq small RNA RNA PCR primer, related to step AB18 Contains the sequence of the Illumina TruSeq Small RNA RNA PCR Primer (RP1).

Table S4. Illumina TruSeq small RNA PCR index primers, related to step AB18 Contains the sequences of Illumina TruSeq Small RNA RNA PCR Index Primers (RPIX).
