# Supplementary material for: Spatial transcriptomics of the nematode Caenorhabditis elegans using RNA tomography
Source: STAR Protoc. 2021 Mar 30;2(2):100411. doi: 10.1016/j.xpro.2021.100411 (PMC8044689; doi:10.1016/j.xpro.2021.100411)
Supplement: Table S3. Illumina TruSeq small RNA RNA PCR primer, related to step AB18 — Contains the sequence of the Illumina TruSeq Small RNA RNA PCR Primer (RP1). [file mmc3.docx]

| **Primer name** | **Sequence** |
| --- | --- |
| RNA PCR primer (RP1) | AATGATACGGCGACCACCGAGATCTACACGTTCAGAGTTCTACAGTCCGA |
