# Supplementary material for: Spatial transcriptomics of the nematode Caenorhabditis elegans using RNA tomography
Source: STAR Protoc. 2021 Mar 30;2(2):100411. doi: 10.1016/j.xpro.2021.100411 (PMC8044689; doi:10.1016/j.xpro.2021.100411)
Supplement: Table S4. Illumina TruSeq small RNA PCR index primers, related to step AB18 — Contains the sequences of Illumina TruSeq Small RNA RNA PCR Index Primers (RPIX). [file mmc4.docx]

| **Index Name** | **Six-Base Sequence in Adapter** | **Six-Base Sequence for Sample Sheet** |
| --- | --- | --- |
| Index 1 (RPI1) | CGTGAT | ATCACG |
| Index 2 (RPI2) | ACATCG | CGATGT |
| Index 3 (RPI3) | GCCTAA | TTAGGC |
| Index 4 (RPI4) | TGGTCA | TGACCA |
| Index 5 (RPI5) | CACTGT | ACAGTG |
| Index 6 (RPI6) | ATTGGC | GCCAAT |
| Index 7 (RPI7) | GATCTG | CAGATC |
| Index 8 (RPI8) | TCAAGT | ACTTGA |
| Index 9 (RPI9) | CTGATC | GATCAG |
| Index 10 (RPI10) | AAGCTA | TAGCTT |
| Index 11 (RPI11) | GTAGCC | GGCTAC |
| Index 12 (RPI12) | TACAAG | CTTGTA |
| Index 13 (RPI13) | TTGACT | AGTCAA |
| Index 14 (RPI14) | GGAACT | AGTTCC |
| Index 15 (RPI15) | TGACAT | ATGTCA |
| Index 16 (RPI16) | GGACGG | CCGTCC |
| Index 17 (RPI17) | CTCTAC | GTAGAG |
| Index 18 (RPI18) | GCGGAC | GTCCGC |
| Index 19 (RPI19) | TTTCAC | GTGAAA |
| Index 20 (RPI20) | GGCCAC | GTGGCC |
| Index 21 (RPI21) | CGAAAC | GTTTCG |
| Index 22 (RPI22) | CGTACG | CGTACG |
| Index 23 (RPI23) | CCACTC | GAGTGG |
| Index 24 (RPI24) | GCTACC | GGTAGC |
| Index 25 (RPI25) | ATCAGT | ACTGAT |
| Index 26 (RPI26) | GCTCAT | ATGAGC |
| Index 27 (RPI27) | AGGAAT | ATTCCT |
| Index 28 (RPI28) | CTTTTG | CAAAAG |
| Index 29 (RPI29) | TAGTTG | CAACTA |
| Index 30 (RPI30) | CCGGTG | CACCGG |
| Index 31 (RPI31) | ATCGTG | CACGAT |
| Index 32 (RPI32) | TGAGTG | CACTCA |
| Index 33 (RPI33) | CGCCTG | CAGGCG |
| Index 34 (RPI34) | GCCATG | CATGGC |
| Index 35 (RPI35) | AAAATG | CATTTT |
| Index 36 (RPI36) | TGTTGG | CCAACA |
| Index 37 (RPI37) | ATTCCG | CGGAAT |
| Index 38 (RPI38) | AGCTAG | CTAGCT |
| Index 39 (RPI39) | GTATAG | CTATAC |
| Index 40 (RPI40) | TCTGAG | CTCAGA |
| Index 41 (RPI41) | GTCGTC | GACGAC |
| Index 42 (RPI42) | CGATTA | TAATCG |
| Index 43 (RPI43) | GCTGTA | TACAGC |
| Index 44 (RPI44) | ATTATA | TATAAT |
| Index 45 (RPI45) | GAATGA | TCATTC |
| Index 46 (RPI46) | TCGGGA | TCCCGA |
| Index 47 (RPI47) | CTTCGA | TCGAAG |
| Index 48 (RPI48) | TGCCGA | TCGGCA |
